# Diagnostic Challenges of Adrenal Venous Sampling for Primary Hyperaldosteronism in a Patient With Subclinical Cushing's Syndrome: A Case Report

**DOI:** 10.1002/ccr3.71578

**Published:** 2025-11-30

**Authors:** Mahsa Malekian, Vahide Sadra, Amir Bahrami, Javad Jalili, Hamidreza Ashayeri

**Affiliations:** ^1^ Endocrine Research Center Tabriz University of Medical Sciences Tabriz Iran; ^2^ Radiology Department, Faculty of Medicine Tabriz University of Medical Sciences Tabriz Iran; ^3^ Student Research Committee Tabriz University of Medical Sciences Tabriz Iran

**Keywords:** adrenal incidentaloma, bilateral idiopathic hyperplasia, mild autonomous hypercortisolism, primary aldosteronism, secondary hypertension

## Abstract

Hypertension is a significant comorbidity in the New World, and its prevalence is rising. Around 5%–10% of cases with hypertension have secondary hypertension. Adrenal gland disease is among the common causes of secondary hypertension. We present a 57‐year‐old male with uncontrolled hypertension and a history of intracranial hemorrhage. The lab evaluation of the patient revealed a K+ of 3.8 mEq/L, Na + of 138 mEq/L, Renin of 2.6 mIU/L, Aldosterone of 47.3 ng/dL, consistent with primary hyperaldosteronism (PHA). The patient's abdominal computed tomography (CT) revealed an adrenal mass measuring 21 mm in the right adrenal gland. However, the adrenal‐vein sampling showed that the mass is probably not the source of aldosterone excess, and a possible diagnosis of adrenal hyperplasia was made. To investigate the adrenal incidentaloma, the 1 mg overnight dexamethasone suppression test was performed. The 8 a.m. cortisol and ACTH levels were reported to be 5 microg/dL and 3.2 pg/mL, and a diagnosis of mild autonomous hypercortisolism was also made. CT is not an accurate method to differentiate between an adrenal‐producing adenoma and bilateral idiopathic adrenal hyperplasia. Even in cases where a visible mass is detected on CT, patients aged 35 or older need to be evaluated for the cause of PHA.

## Key Clinical Messages

- In the setting of hyperaldosteronism, it's essential to biochemically confirm that an incidental adrenal mass is responsible for excess aldosterone secretion because co‐occurrence of pathologies may occur.
- Patients may have more than one cause for secondary hypertension, which may reduce their response to treatment.

## 1 Introduction

Hypertension is estimated to affect more than 1 billion people worldwide, and its prevalence has been increasing in recent years [1]. Adrenal gland diseases are one of the causes of secondary hypertension [2]. Identifying secondary hypertension is essential, as the patients may not respond to the conventional hypertension medication and benefit more from disease‐specific treatments. Here we present a 57‐year‐old male who presented with a history of hypertension and was diagnosed with primary hyperaldosteronism and a unilateral cortisol‐secreting mass.

## 2 Case Presentation and Examination

A 57‐year‐old man with a 20‐year history of hypertension was admitted to the endocrine ward for evaluation of secondary hypertension despite treatment with 160 mg valsartan and 5 mg prazosin, both taken twice a day. His blood pressure during the last year was poorly controlled, and episodes of blood pressure > 180/110 were reported. He also had a history of intracranial hemorrhage 3 years earlier. Additionally, he had three first‐degree relatives with hypertension (father and siblings), but none had a history of adrenal disease. The patient was a non‐smoker and did not complain of snoring, but complained of abdominal pain for 9 months. His blood pressure was 165/95 mmHg with a pulse rate of 73 beats/min. His height was 180 cm, and his BMI was 25 kg/m^2^. There was no tenderness in abdominal examination, and the patient did not have signs of overt Cushing's syndrome.

## 3 Investigations and Treatment

**FIGURE 1:**
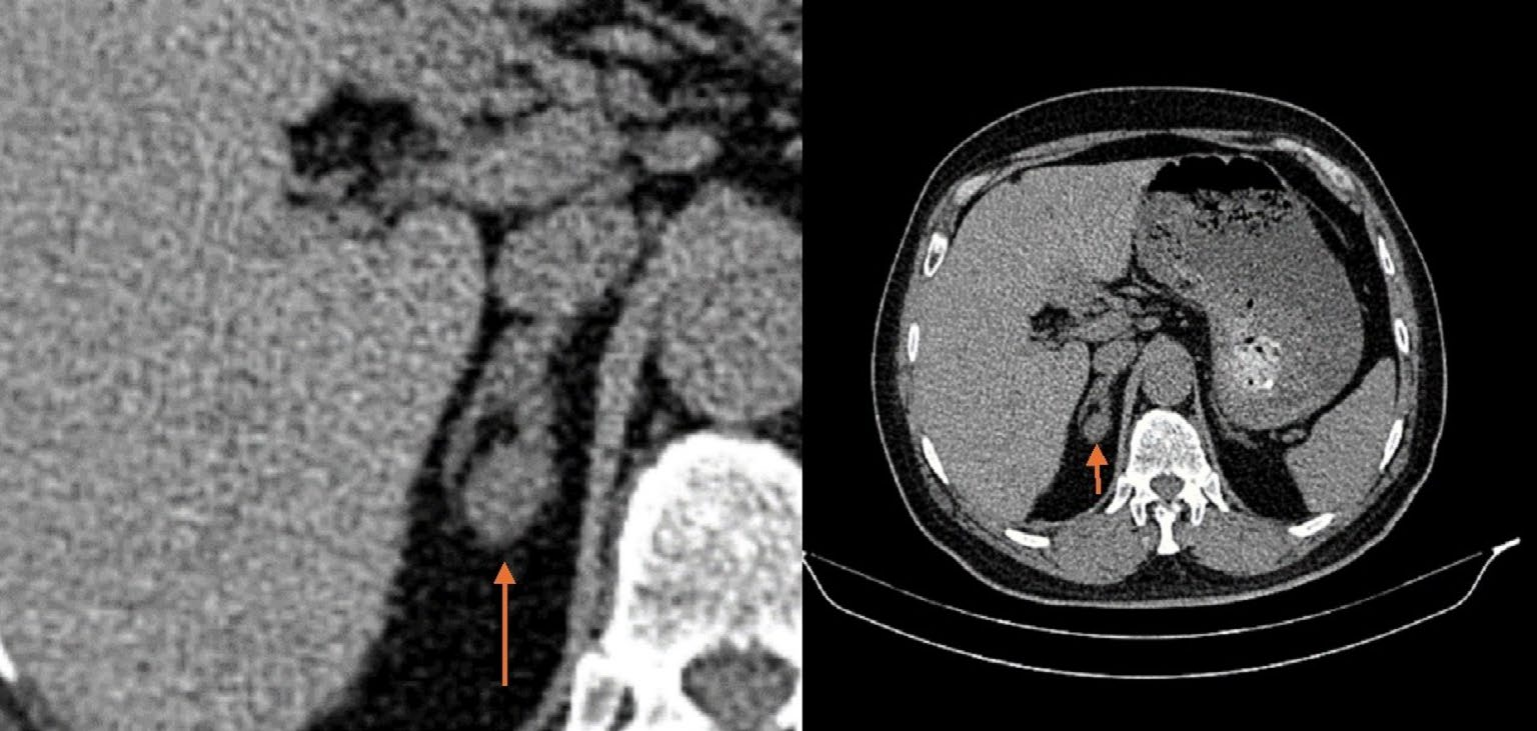
Represents the patient's abdominal CT scan showing a mass in the right adrenal gland (orange arrow).

During the workup for the cause of secondary hypertension, the patient was evaluated for adrenal disease such as primary hyperaldosteronism (PHA). Although valsartan may cause false negative results in the concentration of aldosterone and renin, we decided not to discontinue the antihypertensive agents due to the risk of hypertensive crises and end‐organ damage. Before collecting renin and aldosterone samples, the patient was on a dietary regimen with a daily sodium intake of 3–4 g. The samples were collected at 8:00 a.m., and the patient was positioned upright and seated. The patient's laboratory results revealed potassium of 3.8 mEq/L (normal range: 3.5–4.5), sodium of 138 mEq/L (normal range: 135–145 mEq/L), renin of 2.6 mIU/L (normal range: 5.5–9.9), aldosterone of 47.3 ng/dL (normal range: 3.7–31), fasting blood glucose of 102 mg/dL, and creatinine level of 0.9 mg/dL. The patient had no conditions that could cause a false positive result for the aldosterone and renin measurements, such as non‐steroidal anti‐inflammatory drugs (NSAID), methyldopa, beta‐blockers, or impaired kidney function. A plasma aldosterone concentration > 30 ng/dL, with low renin levels, such as our case, is proposed as diagnostic of PHA and does not need confirmatory tests such as the saline infusion test [3]. In the next step, spiral abdominal computed tomography (CT) without intravenous contrast revealed a 21 mm mass with 29 HU in the right adrenal gland. Figure 1 illustrates the patient's abdominal CT scan.

The patient was diagnosed with PHA and was a candidate for adrenal venous sampling (AVS). Before the procedure, the patient was evaluated for the possibility of pheochromocytoma. He has a 24‐h urine volume of 1800 mL, with a 24‐h urine creatinine of 1300 mg. His 24 h urine metanephrine and normetanephrine levels were 306 and 504 micrograms (normal range: < 400 mcg and < 900 mcg). The urinary tests were suggestive of the absence of pheochromocytoma. The patient was not under any sodium intake restrictions before AVS, and the procedure was performed at 9:00 a.m. We did not use cosyntropin and avoided discontinuing antihypertensive drugs due to the risk of a rise in blood pressure. An expert interventionist performed AVS, and the right AVS was successfully conducted based on the adrenal vein sensitivity index (SI). According to the same ratio, the left AVS seemed to have failed, causing the results to be uninterpretable. It is also noteworthy that the SI of 1.5 was shown not to reduce the specificity of AVS [4]. Still, the cannulization was most likely successful due to the operator's expertise and evaluation during the procedure. In the following lines, we will discuss why the AVS indices are biased in this case.

Results of AVS and its calculated indices are presented in Tables 1 and 2. If we ignore the SI of the left adrenal, the left adrenal was dominant for aldosterone secretion (lateralization ratio (LI): 3.2 and relative aldosterone secretion index (RASI): 5.7). However, there were no signs of right adrenal aldosterone suppression based on the contralateral suppression index (1.49). We hypothesized that the reason for left adrenal dominance was low cortisol levels, either caused by dilution or suppression of cortisol secretion. Either way, the right adrenal nodule was less likely to be the origin of excess aldosterone and PHA. A workup for adrenal incidentaloma was applied further to evaluate the nature of the right adrenal gland mass. The dehydroepiandrosterone‐sulfate (DHEA‐S) level was measured, and the patient underwent a 1 mg dexamethasone suppression test. The DHEA‐S level was 15 μg/dL (normal range: 60–540), and the test revealed an 8 a.m. cortisol level of 5 μg/dL (normal < 1.8) with an ACTH level of 3.2 pg/mL, confirming the diagnosis of mild autonomous cortisol secretion (MACS). The autonomous secretion of cortisol from MACS was identified as the cause of ACTH suppression. The suppression of ACTH caused low cortisol secretion from the left adrenal gland and affected AVS. The excess cortisol secretion in the patient can mimic the biochemical properties of canulization failure and left dominance aldosterone secretion. Either the patient was diagnosed with PHA (most likely, but not definitely due to bilateral adrenal hyperplasia) and MACS. The patient was then discussed about his treatment options and diagnostic challenges faced, and chose medical treatment and follow‐up over surgery. Spironolactone 50 mg twice daily and valsartan 160 mg once daily until his blood pressure became optimal and long‐term follow‐up for signs and symptoms of overt hypercortisolism.

**TABLE 1:** Results of adrenal venous sampling.

Vein	Aldosterone (ng/dL)	Cortisol (microg/dL)	Aldosterone/Cortisol ratio	Aldosterone lateralization ratio
Right adrenal vein	1071	49	21.85	3.82
Left adrenal vein	1170	14	83.57	—
Inferior vena cava	132	9	14.66	—

**TABLE 2:** Indices based on the adrenal venous sampling results.

Index	Result	threshold
Sensitivity index for the right adrenal	5.44	> 2
Sensitivity index for the left adrenal	1.55	> 2
Relative aldosterone secretion index for the right adrenal	1.49	1–2
Relative aldosterone secretion for the left adrenal	5.7	1–2
Lateralization ratio	3.82	> 2
Contralateral suppression index	1.49	< 1

## 4 Follow‐Up and Results

The patient's blood pressure was reported as 140/90 mmHg 1 month after treatment. Since the patient had no symptoms of overt hypercortisolism, a follow‐up was conducted.

## 5 Discussion

PHA or Conn's syndrome is a common cause of secondary hypertension, estimated to be present in 5%–10% of adults with hypertension [5]. Screening for PHA is done by measuring aldosterone and renin levels and the aldosterone‐renin ratio. Multiple drugs such as NSAID, ACE inhibitors/aldosterone receptor blockers, and spironolactone can interfere with the hormonal levels and cause false negative/positive results. However, the test can be performed without the discontinuation of ACE inhibitors/ARB [6]. The discontinuation of other antihypertensive agents is recommended if the results of a prior screening test are negative, but clinical suspicion of PHA is high [6]. Valsartan, an ARB, increases the false negative screening rates, but in our case, despite its use, the screening was positive [6]. We also avoided further confirmatory tests such as saline infusion, since aldosterone levels > 30 and low renin concentration were proposed as diagnostic for PHA [3].

The two most common causes of PHA are adrenal‐producing adenoma (APA) and bilateral adrenal hyperplasia (BAH) [7]. The primary treatment for a unilateral APA is surgery, while the BAH is treated by lifestyle changes and medical management [8, 9]. This difference in treatment strategies necessitates the differentiation. AVS is the best method to differentiate these two diagnoses [10]. The SI is calculated for each adrenal gland to confirm correct cannulation during the AVS. An SI > 1.1–3 without cosyntropin or an SI > 3–5 with cosyntropin stimulation is confirmatory for correct cannulation [11]. The failure usually occurs during the cannulation of the right AVS. In our case, the right SI was 5.44; however, the patient has a cortisol‐secreting mass in the right adrenal that affected the SI in left AVS sampling. The studies have shown that reducing the cut‐off to 1.5 does not reduce the specificity [4]. In our case, the contralateral suppression index increased the suspicion that there was a left adrenal cortisol secretion suppression, affecting LI and RASI. With these, assuming unilateral aldosterone secretion by the left adrenal is questionable, and BAH is most likely.

We propose that the excess cortisol secretion has caused a decrease in ACTH levels and decreased the cortisol secretion from the left adrenal. This suppression of the non‐dominant adrenal has mimicked AVS cannulation failure [12]. The SI is the best method to confirm AVS's diagnostic accuracy. However, our case report implies a situation in which this ratio can be affected. To the best of our knowledge, there is no biochemical test to confirm the accuracy of AVS in the presence of cortisol suppression in only one gland. Some authors have proposed the measurement of DHEA‐S in AVS for aldosterone lateralization, but the clinical significance and appropriate cut‐offs are unclear [13, 14]. Reducing the cut‐off in such cases can also be a solution.

MACS is defined as excessive cortisol secretion without the classic features of overt Cushing syndrome [15]. Some studies suggest that a cortisol level of 1.8 μg/dL after a 1 mg overnight dexamethasone suppression test is an appropriate cutoff for MACS [16]; however, others consider 5 μg/dL to be the cutoff for confirmed MACS [17]. DHEA‐S is also lower in MACS [18]. MACS increases the risk of hypertension, cardiovascular disease, and osteoporosis [15]. Both surgery and medical management are reported to be effective in treating patients with MACS. It has been shown that PHA surgery in the presence of MACS is associated with worse biochemical outcomes [12]. In our case, the patient chose treatment and long‐term follow‐up [19].

## Author Contributions

**Mahsa Malekian:** conceptualization, data curation, formal analysis, investigation, methodology, project administration, resources, supervision, writing – original draft, writing – review and editing. **Vahide Sadra:** investigation, methodology, writing – original draft, writing – review and editing. **Amir Bahrami:** investigation, resources, validation, writing – original draft. **Javad Jalili:** visualization, writing – original draft, writing – review and editing. **Hamidreza Ashayeri:** project administration, software, validation, visualization, writing – original draft, writing – review and editing.

## Funding

The authors have nothing to report.

## Ethics Statement

This manuscript was approved by the Tabriz University of Medical Sciences ethical committee (IR.TBZMED.REC.1403.796). Written informed consent was obtained from the patient to publish the reports and the results of any evaluations and related data.

## Consent

Written informed consent was obtained from the patient to publish any accompanying images.

## Conflicts of Interest

The authors declare no conflicts of interest.

## Data Availability

The datasets supporting the conclusions of this article are included within the article and its additional files.
